# Natural Products in Hemorrhoid Management: A Comprehensive Literature Review of Traditional Herbal Remedies and Evidence-Based Therapies

**DOI:** 10.7759/cureus.83397

**Published:** 2025-05-03

**Authors:** Namdeo Bhagwan Admuthe, Santosh Karajgi, Jyoti Uikey, Navale Mahesh Bhimraj, Ved Prakash, Tejaswini Prasad Babar, Theresa Karra, Ruchita Shrivastava

**Affiliations:** 1 Department of Botany, Annasaheb Awate Arts, Commerce and Hutatma Babu Genu Science College, Pune, IND; 2 Department of Pharmaceutical Quality Assurance, Bijapur Liberal District Education Association’s (BLDEA's) Shri Sanganabasava Mahaswamiji (SSM) College of Pharmacy and Research Centre, Vijayapur, IND; 3 Department of Zoology, Sarojini Naidu Government Girls Post Graduate (Autonomous) College, Bhopal, IND; 4 Department of Botany, Shikshan Prasarak Sanstha’s Sangamner Nagarpalika Arts, Dattatraya Jivanrao Malpani Commerce, and Bhausaheb Narayanrao Sarda Science College, Sangamner, IND; 5 Department of Botany, Bhagat Singh Government Post Graduate College, Ratlam, IND; 6 Department of Pharmacology, Bharati Vidyapeeth (Deemed to be University) College of Ayurved, Pune, IND; 7 Department of Zoology, St. Joseph’s University, Bengaluru, IND; 8 Department of Botany, Government Homescience Post Graduate Lead College, Narmadapuram, IND

**Keywords:** hemorrhoid management, herbal medicine, integrative medicine, medicinal plants, phytotherapy

## Abstract

Hemorrhoids are an anorectal problem that is prevalent and commonly managed with corticosteroids, analgesics, and phlebotonics. These conventional treatments are, however, only temporary in serving relief and come with adverse side effects when used long-term. Alternative herbal remedies, created from traditional medicine with all the evidence of their pharmacological action, promise good results. The effectiveness of various botanicals with the potential of being anti-inflammatory, venotonic, astringent, antioxidant, and wound healing in alleviating hemorrhoidal symptoms is discussed in this review. Therapeutically notable plants such as witch hazel (*Hamamelis virginiana*), horse chestnut (*Aesculus hippocastanum*), and triphala have shown beneficial therapeutic effects on pain, bleeding, and swelling, as well as preventing recurrence. Moreover, Triphala Guggulu and Pilex are polyherbal formulations that offer synergistic benefits in addition to foods rich in polyphenols and the dietary compound, rutin. These promising findings have so far faced challenges related to the standardization, quality control, and clinical validation. The incorporation of these herbal therapies into clinical practice is a well-tolerated holistic approach to the management of hemorrhoids, which requires further investigation to gain evidence-based validation.

## Introduction and background

Hemorrhoids are a common medical issue that affects a large section of the population [1]. They can cause discomfort and lower the quality of life. Herbal therapy has become a viable method for addressing the symptoms and underlying causes of hemorrhoids as people look for holistic alternatives [1]. This article explores the relationship between herbal medicine and hemorrhoid treatment, looking at historical practices and cutting-edge ideas. Around 4.4% of the world’s population is affected by hemorrhoids, which has the highest rate of prevalence at ages 45 to 65 [1,2]. It includes low fiber intake and chronic constipation, along with being sedentary. Options for standard treatments of these conditions are corticosteroids or surgery. They provide only temporary relief and are associated with adverse effects, such as mucosal atrophy and recurrence [2]. Holistic alternatives to modern medicine are herbal medicines based on systems like Ayurveda and Traditional Chinese Medicine. These approaches aim to treat the physiological and systemic causes of hemorrhoids utilizing anti-inflammatory, astringent, and venotonic actions and to support digestive and vascular health by employing natural compounds such as flavonoids, saponins, and mucilage [1,3].

**Figure 1:**
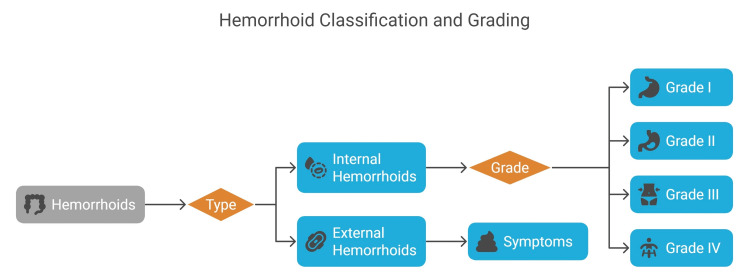
Anatomical classification of hemorrhoids Image Credit: Ruchita Shrivastava

Hemorrhoids are vascular structures located in the anal canal that may swell and inflame for a variety of reasons, including prolonged straining during bowel movements, pregnancy, obesity, and a sedentary lifestyle [2,3]. The symptoms, such as pain during defecation, itching, burning sensation, rectal bleeding, swelling near the anus, and prolapse of hemorrhoidal tissue, affect everyday activities and general well-being [4]. Types of hemorrhoids can be seen in Figure 1. The dentate line determines the two main categories of hemorrhoids, where internal and external types exist. Internal hemorrhoids exist within the rectum, where they stay hidden until they protrude through the anal opening. The extent of prolapse determines each grade from I to IV, with Grade I showing no prolapse and Grade IV being an irreducible condition with possible thrombosis. External hemorrhoids appear beneath the skin near the anus, which leads to pain and swelling along with itching and discomfort during thrombosis [5].

Figure 1 shows the two major types of hemorrhoids that exist as internal and external forms based on their position above or below the dentate line. The rectal canal contains internal hemorrhoids, whereas external hemorrhoids appear below the perianal skin.

Literature search strategy

The literature review analyzed published studies and reviews together with ethnobotanical texts and clinical trials that focused on herbal medicine treatment of hemorrhoids. The research investigated relevant literature from January 2000 to February 2024 through electronic databases, which included PubMed, Scopus, Google Scholar, and ScienceDirect. The research utilized a combination of the keywords “herbal medicine,” “hemorrhoids,” “phytotherapy,” “natural treatment,” “botanical therapy,” “venotonics,” and “anti-inflammatory herbs.” The research team selected articles for review based on their relevance to hemorrhoidal symptom management and phytochemical mechanisms, and traditional use and availability of clinical and observational data. Human applications, as well as preclinical data, which directly translate to clinical scenarios, received priority treatment in the selected literature. Only English-language sources were included.

## Review

Historical use of medicinal plants for hemorrhoid treatment

The use of medicinal herbs for hemorrhoid relief transcends cultural and geographic boundaries, forming an integral part of traditional healing systems. Ancient herbalists and healers recognized the therapeutic potential of plant-based treatments for alleviating symptoms such as bleeding, swelling, and discomfort associated with hemorrhoids. In Ayurvedic medicine, botanicals like neem (*Azadirachta indica*) and haritaki (*Terminalia chebula*) were employed for their potent anti-inflammatory and astringent properties, contributing to improved bowel regularity and local symptom relief [1]. Traditional Chinese Medicine (TCM) also utilized herbs such as horse chestnut (*Aesculus hippocastanum*) to enhance venous circulation, reduce vascular congestion, and soothe hemorrhoidal inflammation [2].

Scientific basis of herbal remedies (active compounds and mechanisms)

Recent pharmacological studies have elucidated the bioactive constituents and physiological mechanisms underlying the effectiveness of herbal treatments for hemorrhoids. For example, witch hazel (*Hamamelis virginiana*) contains astringent tannins that constrict blood vessels, reduce local inflammation, and alleviate minor bleeding and irritation [3]. Similarly, horse chestnut seed extract is rich in aescin, a saponin with venotonic and anti-inflammatory properties that promote vascular tone and diminish venous congestion, leading to reduced swelling and discomfort in patients with venous insufficiency and hemorrhoids [4].

Modern pharmacological evaluations have demonstrated that horse chestnut extract containing aescin exhibits clinical efficacy comparable to synthetic flavonoid therapies in improving venous tone and reducing perianal edema [6]. Butcher’s broom (*Ruscus aculeatus*), through the activation of alpha-adrenergic receptors, enhances vascular resistance and promotes lymphatic drainage, leading to reduced perianal swelling and discomfort in hemorrhoidal patients. Witch hazel, with its high tannin content, acts as a natural astringent that helps reduce bleeding and irritation by stabilizing the capillary walls and decreasing vascular permeability [7]. These herbs act across multiple biological pathways, including anti-inflammatory, antioxidant, and vasoprotective mechanisms, offering a broader therapeutic effect than corticosteroids and with a significantly lower incidence of adverse effects [2,6].

Comparative effectiveness of herbal and conventional therapies

Conventional medications, including corticosteroids, analgesics, and phlebotonics such as diosmin-hesperidin, are commonly prescribed for the management of hemorrhoids. While these pharmacological agents provide rapid symptom relief, they are associated with notable side effects such as mucosal thinning, skin atrophy, dependency, and gastrointestinal discomfort when used long-term. In contrast, botanicals like *Ruscus aculeatus*, *Aesculus hippocastanum*, and *Calendula officinalis* exhibit multi-targeted actions, modulating inflammation, enhancing venous tone, and promoting tissue regeneration with a more favorable safety and tolerability profile [6,7].

A meta-analysis comparing herbal venotonics with conventional synthetic agents found that natural compounds provided comparable, and in some cases superior, relief from symptoms associated with Grade I-II hemorrhoids [1,6]. These herbal interventions were associated with better patient satisfaction, reduced recurrence rates, and improved adherence, likely due to their reduced side effect profile and holistic approach to symptom management.

Investigating the herbal approach from a holistic angle

Herbal medication provides holistic treatment for hemorrhoids because it targets symptoms and root causes simultaneously. The natural therapeutic properties of medicinal herbs enable herbal therapies to perform three functions: reducing inflammation and improving blood circulation while supporting healthy bowel function [3]. The natural health approach matches basic holistic principles that appeared first in herbal medicine practices. Herbal therapies functioned as a complete system with bodily balance in traditional holistic healthcare approaches. Herbal selection happens based on both medicinal properties and energetic effects, such as warming and drying. Triphala offers multiple benefits, including digestive support, liver detoxification, and relief from defecation straining, which are essential for hemorrhoid treatment [5]. These therapeutic methods support the practices by focusing on stress reduction and circulation enhancement, and bowel health maintenance through practices of yoga and mindfulness, and plant-based dietary approaches.

Common medicinal plants and clinical applications

The ability of medicinal plants to reduce the pain and symptoms connected with hemorrhoids has long been revered. The following section focuses on three notable herbs renowned for their potency in the treatment of hemorrhoids:

Witch Hazel (Hamamelis virginiana): Astringent and Anti-inflammatory Properties

Witch hazel, derived from the leaves and bark of *Hamamelis virginiana*, has been widely used in traditional and modern medicine due to its potent astringent and anti-inflammatory properties. The tannins present in witch hazel help constrict blood vessels, reduce capillary permeability, and minimize local irritation, making it especially effective for anorectal conditions. Several studies and clinical guidelines have recognized the use of witch hazel for symptom relief in hemorrhoidal disease. In particular, topical witch hazel preparations such as medicated pads and ointments have demonstrated efficacy in reducing anal itching, mild bleeding, burning, and inflammation in cases of external or Grade I-II internal hemorrhoids [2]. According to the American Society of Colon and Rectal Surgeons (ASCRS) and referenced patient care resources, witch hazel is a commonly recommended over-the-counter (OTC) remedy for conservative hemorrhoid management. Its availability in multiple topical forms makes it a convenient and well-tolerated option for symptomatic relief [5].

Butcher’s Broom (Ruscus aculeatus): Venotonic Effects and Improved Circulation

Butcher’s broom, a traditional European medicinal herb, has gained recognition for its venotonic and anti-inflammatory properties, particularly in the treatment of vascular disorders. The primary active constituents, ruscogenins, act by constricting venous vessels and enhancing microcirculation [6]. These pharmacological actions make *Ruscus aculeatus* a suitable agent for treating conditions characterized by venous insufficiency, including hemorrhoids. Clinical investigations have demonstrated that butcher’s broom, when administered orally or topically, can alleviate symptoms such as perianal pain, swelling, and discomfort in patients with early-stage hemorrhoids. In one randomized study, patients receiving a ruscogenin-based formulation reported significant reductions in edema, bleeding episodes, and the need for analgesics compared to placebo groups. Its use is also supported in combination therapies with hesperidin or diosmin in conservative hemorrhoidal treatment regimens [4,8].

Horse Chestnut (Aesculus hippocastanum): Anti-inflammatory and Venotonic Benefits

Horse chestnut seeds are a popular alternative for treating hemorrhoids and venous insufficiency because of their anti-inflammatory and venotonic properties. An important component of horse chestnut, aescin, has anti-inflammatory qualities that can lessen hemorrhoid swelling and irritation [4]. Additionally, it has venotonic properties that improve blood flow and lessen venous congestion, relieving symptoms. Trials have indicated that standardized horse chestnut extract can significantly improve symptoms such as itching, swelling, and pain in hemorrhoidal conditions [6].

These typical medicinal herbs are a collection of natural cures that may supplement established methods of treating hemorrhoids. Before using these remedies, as with any herbal treatment, it is advisable to speak with a healthcare provider, especially if you have underlying medical concerns or are taking medication.

Soothing solutions (herbal topical applications)

With the progression of pharmaceutical delivery systems, topical herbal applications have progressed as well. For instance, liposomal aloe vera increases mucosal absorption and provides for long-lasting anti-inflammatory action [8]. Triterpenoids from calendula ointments promote angiogenesis and fibroblast activity and speed up wound healing [7]. Apigenin, a bioflavonoid with proven anti-inflammatory and antispasmodic properties, makes chamomile compresses soothing to irritated areas [9]. Due to these gentle, natural, and noninvasive effects, these options are often preferred by patients. Herbal topical applications offer gentle and soothing relief for hemorrhoid discomfort. The following remedies harness the natural properties of plants to provide comfort and promote healing:

Calendula (Calendula officinalis) Creams and Ointments

Calendula, renowned for its anti-inflammatory and wound-healing properties, is a popular choice for topical application. Creams and ointments infused with calendula extract can help reduce irritation, redness, and itching associated with hemorrhoids. The plant's flavonoids and triterpenoids contribute to its soothing effects, making it a valuable addition to a comprehensive hemorrhoid care regimen [5]. Studies have confirmed improved healing rates and reduced inflammation in anal fissures and hemorrhoidal wounds with calendula-based preparations.

Aloe Vera (Aloe barbadensis) Gel

Derived from the succulent leaves of the *Aloe barbadensis* plant, aloe vera gel is cherished for its cooling and anti-inflammatory effects. When applied topically, aloe vera gel can provide relief from pain, burning, and itching caused by hemorrhoids. Its mucilaginous components help soothe inflamed tissues while promoting healing [7]. Clinical studies demonstrate that aloe preparations improve pain scores and healing time when used in combination with standard hemorrhoidal therapies [10].

Chamomile (Matricaria chamomilla) Compresses and Soaks

Chamomile, a gentle and calming herb, offers comfort through compresses and soaks. Infusing warm water with chamomile flowers creates a soothing solution that can be used for sitz baths or compresses. Chamomile's anti-inflammatory and antioxidant properties help alleviate discomfort and promote relaxation, offering respite from hemorrhoidal symptoms [8]. Evidence suggests a reduction in inflammation and faster resolution of itching and irritation with consistent chamomile use.

Topical and internal herbal therapies

Herbal remedies taken internally contribute to overall digestive health and can play a role in managing hemorrhoids. The following internal remedies address underlying factors that contribute to hemorrhoid development:

Psyllium (Plantago ovata): Fiber for Improved Bowel Regularity

Psyllium, rich in soluble fiber, is known to promote bowel regularity and soft, well-formed stools. Adequate fiber intake can reduce the strain associated with bowel movements, helping prevent exacerbation of hemorrhoids. Psyllium supplements or husk can be easily incorporated into the diet to support digestive health and alleviate constipation [9]. Clinical trials indicate reduced straining and frequency of bleeding episodes with daily psyllium supplementation [4].

Triphala: Traditional Ayurvedic Blend for Digestive Health

Triphala, an Ayurvedic formulation comprising three fruits (Amalaki, Bibhitaki, and Haritaki), offers comprehensive digestive support. Its mild laxative properties aid in maintaining regular bowel movements, reducing the likelihood of straining during defecation. Triphala's antioxidant and anti-inflammatory effects contribute to overall gastrointestinal well-being [10]. Evidence supports its use in improving bowel function and alleviating discomfort in hemorrhoid patients [5].

Rutin-Rich Foods and Supplements: Strengthening Blood Vessel Walls

Rutin, a flavonoid found in certain foods and herbs, supports vascular health by strengthening blood vessel walls. Incorporating rutin-rich foods like citrus fruits, berries, and buckwheat into the diet can contribute to enhanced blood vessel integrity and reduced hemorrhoidal discomfort [11]. Clinical data show that rutin supplementation can lead to a measurable decrease in bleeding, pain, and perianal pressure associated with hemorrhoids [8].

These internal herbal remedies offer a holistic approach to managing hemorrhoids by addressing dietary and digestive aspects that contribute to symptom relief.

Table 1 shows commonly used herbal treatments for hemorrhoids, together with their therapeutic benefits and reported therapeutic results, and specific applications. The table integrates both traditional knowledge with clinical research findings, which demonstrate how these remedies decrease hemorrhoidal symptoms, including bleeding and inflammation, pain, and venous congestion. 

**Table 1:** Herbal medicines used in the treatment of hemorrhoids with clinical and traditional evidence RCT: randomized controlled trials

Herbal Medicine	Properties and Benefits	Application	Treatment Evidence	Reference
Witch hazel (*Hamamelis virginiana*)	Astringent and anti-inflammatory properties help reduce swelling and irritation.	Topical creams, ointments, and medicated pads for external application.	Clinical use shows reduced bleeding, irritation, and swelling in mild hemorrhoids.	[7]
Butcher's broom (*Ruscus aculeatus*)	Venotonic effects improve blood circulation and reduce venous congestion.	Oral supplements or topical creams containing butcher's broom extract.	Associated with decreased perianal pain and edema in early-stage hemorrhoid cases.	[6]
Horse chestnut (*Aesculus hippocastanum*)	Anti-inflammatory and venotonic properties alleviate discomfort and enhance blood flow.	Oral capsules, creams, or ointments with horse chestnut extract.	RCTs show improvement in venous tone and reduced discomfort in hemorrhoid patients.	[6,11]
Calendula (*Calendula officinalis*)	Anti-inflammatory and wound-healing properties soothe irritation and promote healing.	Creams, ointments, or balms for topical application.	Supports wound healing and reduces inflammation in anorectal applications.	[7]
Aloe vera (*Aloe barbadensis*)	Cooling and anti-inflammatory effects provide relief from pain and itching.	Pure aloe vera gel is applied topically or included in creams and ointments.	Shown to relieve pain and burning in hemorrhoids; enhances mucosal repair.	[10]
Chamomile (*Matricaria chamomilla*)	Anti-inflammatory and antioxidant properties offer calming relief.	Sitz baths, compresses, or chamomile-infused creams for external use.	Provides comfort and reduces irritation; used effectively in sitz baths.	[9]
Psyllium (*Plantago ovata)*	Soluble fiber improves bowel regularity and reduces straining.	Oral supplements or psyllium husk mixed with water or food.	Improves stool consistency and reduces bleeding and straining.	[4]
Triphala	Ayurvedic blend supports digestive health and regulates bowel movements.	Oral powder, capsules, or tablets as per Ayurvedic guidelines.	Promotes bowel regularity, reducing straining and recurrence risk.	[5]
Rutin-rich foods and supplements	Strengthens blood vessel walls and supports vascular health.	Incorporate rutin-rich foods (citrus fruits, berries) or dietary supplements.	Strengthens vascular walls, decreasing bleeding and pain symptoms.	[8]
Witch hazel compresses	Soothes inflammation, reduces itching, and promotes healing.	Compresses soaked in witch hazel solution are applied to the affected area.	Helps soothe inflamed tissues and reduces itching and burning.	[7]
Plantain (*Plantago major*)	Anti-inflammatory properties and mucilage content provide relief from itching and discomfort.	Topical application of plantain leaf poultices or creams.	Used to relieve itching and promote comfort due to mucilage content.	[7]
Yarrow (*Achillea millefolium*)	Astringent and anti-inflammatory effects help reduce swelling and discomfort.	Sitz baths or yarrow-infused oils for external application.	Traditional use supports reduced swelling and better local circulation.	[7]
Gotu kola (*Centella asiatica)*	Promotes collagen synthesis and tissue repair, aiding in healing.	Topical creams or ointments containing gotu kola extract.	Aids in tissue regeneration; accelerates wound repair.	[11]
Witch hazel suppositories	Direct application for internal relief, reducing inflammation and discomfort.	Herbal suppositories containing witch hazel extract.	Applied to reduce inflammation and discomfort in internal hemorrhoids.	[7]
Marshmallow root (*Althaea officinalis*)	Soothing mucilage content eases irritation and supports healing.	Topical creams, poultices, or herbal compresses.	Used topically to soothe and protect inflamed tissues.	[10]
Comfrey (*Symphytum officinale*)	Contains allantoin, aiding in tissue repair and reducing irritation.	Comfrey-infused oils or ointments for external use.	Promotes epithelial healing and reduces irritation.	[7]
St. John's Wort (*Hypericum perforatum*)	Anti-inflammatory and analgesic properties offer relief from pain and discomfort.	St. John's Wort oil or ointment for external application.	Used to alleviate localized pain and discomfort.	[9]
Neem (*Azadirachta indica*)	Antimicrobial and anti-inflammatory effects support healing and hygiene.	Neem oil or creams for external use, or neem leaf baths.	Traditional remedy for healing and cleansing hemorrhoidal tissues.	[5]
Frankincense (*Boswellia serrata)*	Anti-inflammatory and wound-healing properties promote tissue repair.	Topical creams or oils containing Frankincense extract.	Aids in reducing inflammation and supports tissue regeneration.	[2]

Synergistic herbal formulations

Complementary plant compound mechanisms produce synergistic effects in herbal medicine when physicians prescribe combination therapy treatments. Ayurvedic medicines like Triphala Guggulu and Pilex that contain neem (*Azadirachta indica*) together with haritaki (*Terminalia chebula*) and lajjalu (*Mimosa pudica*) provide a wide range of therapeutic benefits, which include stool regulation, systemic detoxification, and wound healing enhancement [5]. Clinical research shows that multi-herbal preparations surpass single-herbal extracts because of their combined pharmacological activities from flavonoids, tannins, and saponins in these combinations [1,5]. Laboratory studies show that medical formulations that unite aloe vera with horse chestnut (*Aesculus hippocastanum*) exhibit stronger benefits than individual components, as they treat symptoms better and lower recurrence probability [6,10]. Research evidence validates the use of herbal combinations as an effective approach for treating hemorrhoids in healthcare protocols.

Role of antioxidants and polyphenols

Research investigations now show that oxidative stress plays an essential role in the development of hemorrhoidal inflammation and tissue breakdown. The polyphenolic compounds rutin and epicatechin show effectiveness in reactive oxygen species (ROS) neutralization, which strengthens blood vessel structure and reduces capillary weakness [8]. Blood vessel wall tensile strength increases while hemorrhoidal bleeding decreases when patients consume antioxidants due to their protective properties. Patients who incorporate polyphenol-rich foods, especially blueberries with grapes, and green tea, into their diet might experience supplemental benefits with their herbal treatment. The antioxidant effects of certain rituals face limitations because vitamin C and E depletion occur from fasting or heat exposure, which reduces their therapeutic benefits. The clinical research shows that patients with Grade I and II hemorrhoids experience reduced anal pressure and decreased rectal bleeding and pain intensity after receiving rutin supplementation [8,9].

Patient-centered benefits and compliance

Patients prefer herbal remedies as their choice for natural and sustainable alternative treatments compared to traditional hemorrhoid therapies. Medical research activities focused on patient needs demonstrate that herbal medications show better patient satisfaction through better tolerability, along with lifestyle modification interventions consisting of adequate hydration and physical activity, and increased fiber consumption [3,4]. Different forms of herbal products, such as creams and gels and teas, and capsules, allow patients to customize their use according to symptom intensity and personal choice. Patients who use these accessible herbal treatments for chronic or recurrent hemorrhoids experience better adherence and quality of life because they have fewer adverse effects.

Future directions and innovations

The process of standardization and safety profiling and dosage precision, along with safety profiling for herbal treatments, remains a scientific challenge. The combination of nano-encapsulation systems is expected to transform the bioavailability of aloe vera and witch hazel active agents by developing sustained release and targeted delivery methods [8]. Plant synergy modeling with artificial intelligence represents a promising tool for pharmacological optimization because it can identify effective combinations along with precise dosing strategies when multiple herbs are used together. The integration of herbal medicine with conventional therapeutic approaches should have formal clinical testing to boost the scientific support behind combined strategies for treatment [1,3]. New digital health applications allow patients and medical practitioners to access personalized herb-drug interaction features and advisory systems, which support safe health choices about herbal medicines.

Collaborative research for advanced herbal solutions

Scientific breakthroughs in herbal hemorrhoid therapies require professionals from herbalists and pharmacologists, and clinical research backgrounds to work together. The development of safe and effective novel formulations becomes possible through the unification of traditional knowledge with strict scientific methodologies. The scientific community must invest efforts into pharmacokinetic profiling and bioactive standardization alongside conducting large-scale randomized clinical trials to strengthen proof of therapeutic effects.

Education and awareness (empowering patients and healthcare professionals)

The acceptance of herbal medicine demands both societal focus on education and patient safety practices to create safe medical use possibilities. Patients obtain better results when healthcare providers provide them with precise, evidence-based information about medication dosages and formulations, together with herb-drug interaction warnings. The integration of integrative care practices depends on healthcare professionals maintaining current knowledge, together with physicians, naturopaths, and pharmacists. Medical education programs should integrate herbal medicine knowledge to create patient-focused healthcare models that unite conventional and complementary treatments for hemorrhoid care.

## Conclusions

Herbal medicine proves itself as both a scientifically validated and traditional treatment option for hemorrhoids, thanks to increasing evidence from research. Multiple botanical agents, including witch hazel, butcher's broom, horse chestnut, calendula, aloe vera, and triphala, demonstrate anti-inflammatory along with venotonic, and astringent effects that help treat both hemorrhoid symptoms and the pathophysiological causes of venous congestion, oxidative stress, and bowel irregularity. Clinical observations, together with traditional wisdom and combined internal and topical applications, strengthen the therapeutic potential of these agents to reduce pain, bleeding, swelling, and recurrence. Herbal combinations with polyphenol content enhance treatment results, and patients tend to follow these treatments because they contain natural ingredients that are accessible and comfortable for use. The complete implementation of these agents into clinical practice needs standardized approaches and effective dosing methods, and research-based evidence integration, which can be achieved through additional pharmacological studies and clinical trials. Innovations, including nano-encapsulation, along with AI-based plant synergy modeling and herb-drug interaction databases, have the potential to improve both the safety and precision of phytotherapeutic treatments. Herbal medicine maintains its promise to transform into a novel, sustainable healthcare method that goes beyond adding therapeutic value to hemorrhoid treatment.
